# Individuals with mild-to-moderate hip osteoarthritis have lower limb muscle strength and volume deficits

**DOI:** 10.1186/s12891-018-2230-4

**Published:** 2018-08-21

**Authors:** Aderson Loureiro, Maria Constantinou, Laura E. Diamond, Belinda Beck, Rod Barrett

**Affiliations:** 1 Menzies Health Institute Queensland, School of Allied Health Sciences, Griffith University, Gold Coast, QLD 4222 Australia; 2 Pontifical Catholic University (PUCRS), Porto Alegre, Brazil; 3 University of Rio dos Sinos (UNISINOS), São Leopoldo, Brazil; 4 Australian Catholic University, Brisbane, QLD 4014 Australia; 5 Centre of Clinical Research Excellence in Spinal Pain, Injury & Health, School of Health & Rehabilitation Sciences, The University of Queensland, Brisbane, QLD Australia

**Keywords:** Atrophy, Weakness, OA, MRI, Isometric

## Abstract

**Background:**

Individuals with advanced hip osteoarthritis (OA) exhibit generalized muscle weakness of the affected limb and so clinical practice guidelines recommend strength training for the management of hip OA. However, the extent and pattern of muscle weakness, including any between-limb asymmetries, in early stages of the disease are unclear. This study compared hip and knee muscle strength and volumes between individuals with mild-to-moderate symptomatic and radiographic hip OA and a healthy control group.

**Methods:**

Nineteen individuals with mild-to-moderate symptomatic and radiographic hip OA (*n* = 12 unilateral; *n* = 7 bilateral) and 23 age-matched, healthy controls without radiographic hip OA or hip pain participated. Isometric strength of the hip and knee flexors and extensors, and hip abductors and adductors were measured. Hip and thigh muscle volumes were measured from lower limb magnetic resonance images. A full-factorial, two-way General Linear Model was used to assess differences between groups and between limbs.

**Results:**

Participants in the hip OA group demonstrated significantly lower knee flexor, knee extensor, hip flexor, hip extensor and hip abductor strength compared to controls and had significantly lower volume of the adductor, hamstring and quadriceps groups, and gluteus maximus and gluteus minimus muscles, but not tensor fasciae latae or gluteus medius muscles. There were no between-limb strength differences or volume differences within either group.

**Conclusions:**

Atrophic, bilateral hip and knee muscle weakness is a feature of individuals with mild-to-moderate hip OA. Early interventions to target muscle weakness and prevent the development of strength asymmetries that are characteristic of advanced hip OA appear warranted.

## Background

People with hip osteoarthritis (OA) often experience joint pain, stiffness, reduced joint range of motion, and muscle weakness [1–4]. These deficits can limit performance of activities of daily living and diminish quality of life [5]. Hip OA has no cure, and progression to more advanced disease occurs in many patients. Conservative non-pharmacological interventions focus primarily on alleviating pain and improving function [6–11]. Individuals with advanced hip OA exhibit generalized muscle weakness of the affected limb [12–19], which is underpinned by a combination of muscle atrophy [16, 18, 20–22], reduced muscle density [14, 21, 22], and muscle inhibition [22]. Clinical practice guidelines recommend land-based therapeutic exercise for the management of hip OA [23], most notably resistance training, which can reduce pain, stiffness and self-reported disability, and improve strength, physical function and joint range of motion [24, 25]. At present however, there is limited understanding of the extent and pattern of muscle weakness in earlier stages of the disease. If muscle weakness were also found to be a feature of mild-moderate hip OA, then interventions such as resistance training that target muscle weakness and prevent the development of strength asymmetries characteristic of advanced hip OA [26] may be warranted in earlier stages of the disease.

Most investigations of muscle properties in hip OA have included individuals in advanced stages of the disease [14, 16, 18, 20–22]. Studies that included patients across the early spectrum of disease severity [12, 27] reported lower gluteal muscle volumes in individuals with unilateral hip OA compared to their contralateral side and a group of healthy controls. Deficits in hip abduction and internal rotation strength of the affected leg compared to healthy controls were also noted and suggest that muscle weakness could also be a feature of earlier stages of the disease than previously reported. It therefore remains unclear whether muscle weakness and atrophy that precede advanced stages of the disease extend beyond the abductor muscle group of the affected leg to other prime movers (i.e. quadriceps, hamstrings, adductors) within the most affected leg or the contralateral leg. Evidence of between-limb differences in hip and knee muscle strength and/or muscle volume have been reported in advanced hip OA [12, 22] and following total hip replacement [21]. While Grimaldi et al. [20, 28] reported an absence of asymmetry in the volume of the gluteal, piriformis, and tensor fascia latae muscles in mild hip OA, symmetry of other important hip and knee muscles is yet to be assessed. An improved understanding of whether muscle weakness and atrophy in mild-to-moderate hip OA is generalized or specific to certain muscles or muscle groups in the lower extremity is required to appropriately inform and optimise management programs.

The purpose of this study was to compare hip and knee muscle strength and volumes between individuals with mild-to-moderate symptomatic and radiographic hip OA and a healthy control group. Based on evidence from studies which report muscle weakness and atrophy in knee OA [29], it was hypothesized that individuals with mild-moderate hip OA would similarly exhibit muscle weakness and lower limb muscle atrophy, particularly in their (more) affected limb, compared to healthy age-matched controls.

## Methods

### Participants

Individuals aged 45 to 80 years with symptomatic unilateral or bilateral hip osteoarthritis were recruited from local hospital orthopaedic waiting lists to participate in this case-control study. Healthy controls were recruited through advertising and word-of-mouth. All participants were screened through radiographic examination (anterior-posterior radiographs of the pelvis and hips) and self-reported measures of pain and function (modified Harris Hip Score (HHS) [30]). Unilateral and bilateral hip OA participants were required to have hip pain and/or functional limitations during activities of daily living (HHS ≤ 95; 0 = extreme hip problems, 100 = no hip problems) and had a Kellgren-Lawrence (KL) grade [31] for their affected hip(s) of 2 or 3 and/or joint space width (JSW) ≤ 3 mm). Unilateral hip OA participants had KL scores of 0 or 1 for their contralateral hip. Healthy controls were required to have no hip pain or functional limitations during activities of daily living (HHS > 95) and had KL grades ≤1 and JSW > 3 mm for both hips. KL scores were determined by a single radiologist in a blinded manner from bilateral weight-bearing radiographs performed in 15 degrees of femoral internal rotation [32]. The same radiologist electronically measured supero-medial, apical and supero-lateral hip JSW [33]. Exclusion criteria for both groups included: (i) previous lower limb or back fracture or surgery; (ii) history of trauma to the hip joint or pelvis region; (iii) other forms of arthritis, diabetes, cardiac or circulatory conditions; and (iv) use of corticosteroid medication. All individuals were able to walk without physical assistance or devices.

An a priori power analysis using hip abduction strength data from Zacharias et al. [27] (hip OA = 0.15(0.09); controls = 0.25(0.10)) estimated a minimum of 12 participants were required in each group (significance level was set at *α*= 0.05 and power at 0.80 (one tail)). Participants were enrolled concurrently in another study [34]. This study was approved by the institutional Human Research Ethics Committee and written informed consent was obtained from the participants prior to participating in the study.

### Procedures

Participants initially attended a laboratory session to assess bilateral isometric strength of the lower extremity muscles. Anthropometric measures including height (m) and body mass (kg) were also taken. Body mass index (BMI) was determined as weight divided by the square of height (kg/m^2^). Within 48 h of attending the strength testing session, participants underwent bilateral magnetic resonance imaging (MRI) of their lower extremity in a private radiologic clinic. This study conformed to the STROBE statement for reporting case-control studies [35].

Maximal voluntary isometric hip and knee muscle strength was measured using an isokinetic dynamometer (Biodex System 4, Biodex Medical Systems, USA) using a protocol adapted from Carty et al. [36]. Hip flexor, extensor, adductor and abductor strength were assessed while standing in 0° of hip flexion and adduction (neutral position), with the knee constrained in 60° of flexion using a post-surgical orthopaedic knee brace, and the ankle in 5° of plantar flexion. Participants were allowed to apply a light force against the dynamometer head for the purpose of maintaining balance. Knee flexor and extensor strength tests were performed while seated. Knee flexor strength was assessed at 30° of knee flexion with the hip in 90° of flexion and the ankle in 5° of plantar flexion. Knee extensor strength was assessed at 60° of knee flexion with the hip in 70° of flexion and the ankle in 5° of plantar flexion. The order of strength measurements was from hip to knee randomized by limb. Participants performed a 5-s practice trial at 75% of maximal effort for each exercise, followed by a 60-s rest and a 5-s maximal contraction. Prior to each maximal effort trial, participants were instructed to contract as hard as they could for 5-s, with verbal encouragement provided to help maximize effort. The instantaneous peak isometric torque for each exercise was adjusted to account for the torque due to the dynamometer attachment and lower limb segments distal to the joint being tested in accordance with the recommendations of Kellis and Baltzopoulos [37], using body segment parameters estimated from Dempster [38]. Isometric strength at each joint, in each direction, was defined as the peak torque measured normalized to body mass (Nm/kg).

**Fig. 1:**
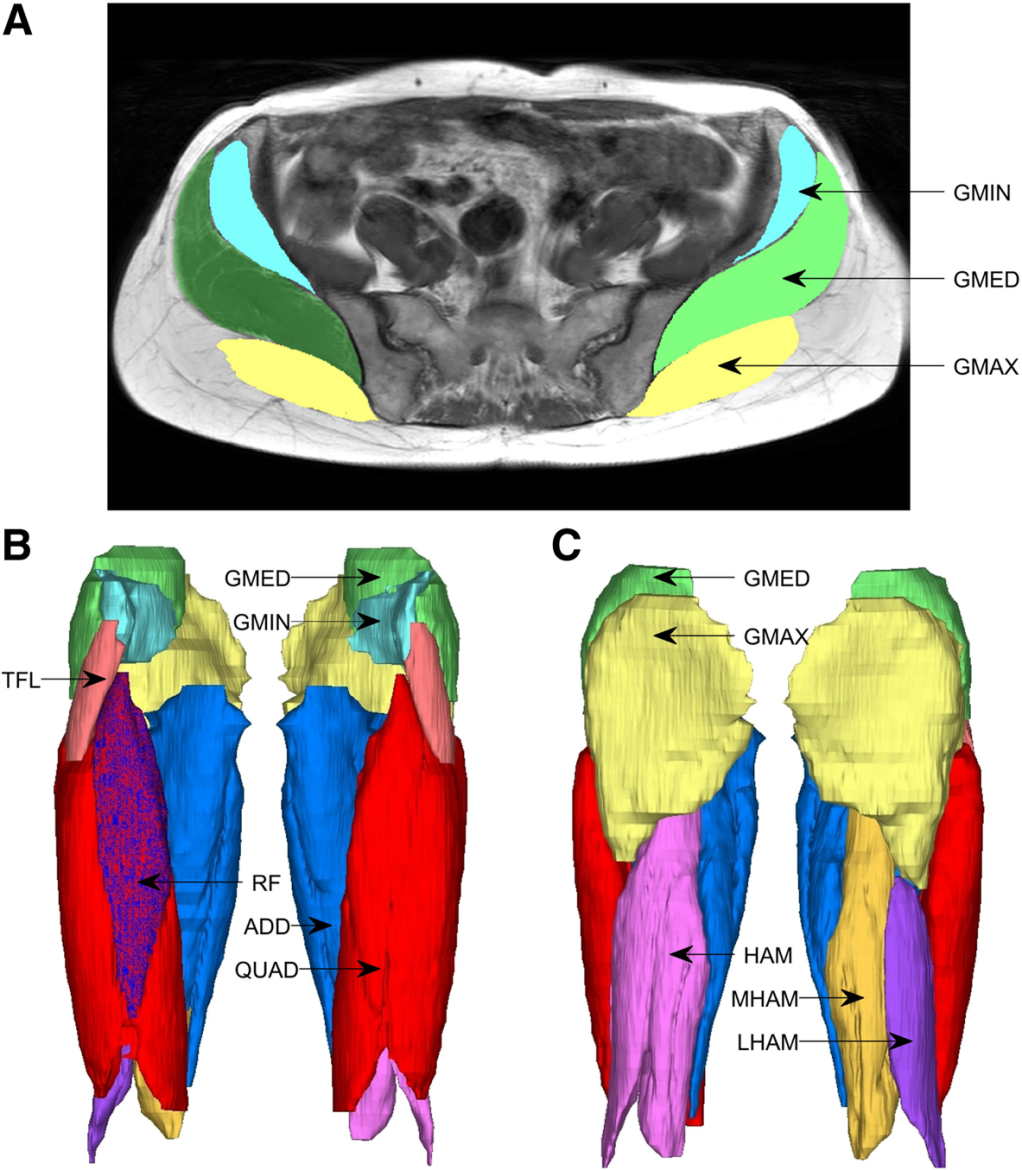
Muscle and muscle group segmentation from magnetic resonance images of a representative healthy control participant; **a** superior view of muscle masks segmented from an individual transverse plane slice; **b**-**c** anterior and posterior views, respectively, of 3D rendering of thigh and hip muscles (GMIN-gluteus minimus; GMED-gluteus medius; GMAX-gluteus maximus; TFL-tensor fasciae latae; ADD-adductors; QUAD-quadriceps; HAM-hamstrings)

A 3.0 T MRI whole body scanner (Phillips Ingenia, Phillips Medical, Netherlands), was used to image bilateral lower limbs of all participants. Axial plane scans were performed with participants positioned supine in the scanner using body coil arrays placed superiorly on the limbs with contiguous slices taken from approximately 2-cm superior to the iliac crest to approximately 2-cm inferior to the proximal tibio-fibula joint. Both lower limbs were scanned simultaneously with T1 weighted 2-dimensional gradient-recall acquisition in the steady state; slice thickness 10-mm, inter-slice gap 1-mm, flip angle 90^0^; repetition time 677 msec, echo time 6.5 msec; field of view 280 × 500 × 219 mm; 352 × 499-pixel matrix; acquisition time 1 min 29 s. Volumes of individual muscles (tensor fasciae latae (TFL), gluteus maximus (GMax), gluteus medius (GMed), gluteus minimus (GMin)) and muscle groups (adductors (i.e. magnus, gracilis, brevis, and longus) (Add), quadriceps (i.e. vastus medialis, vastus intermedius, vastus lateralis, rectus femoris) (Quad), hamstrings (i.e. semimembranosus, semitendinosus, biceps femoris) (Hams)) were then calculated using Mimics software (Materialise N.V., Belgium). The ilopsoas muscle was not assessed as it was only partially visible in the MRI scans obtained. Muscles were segmented on a slice-by-slice basis by a single reader (AL) using the semi-automated lasso tool (Fig. 1a). These data were then combined to create the final 3-dimensional (3D) rendering. The 3D-volume object was wrapped using finest detail of 0.50 mm and a gap closing distance of 1.00 mm, followed by a smoothing process with a factor of 1.0 and 4 iterations. Finally, the muscle volumes were determined by summing all pertinent pixels within the resultant binary volume (Fig. 1b-c). Individual and group muscle volumes were normalized to body mass (cm^3^/kg). Reliability of muscle segmentation was assessed following the approach described by Grimaldi et al. [20]. In brief this involved the same investigator (AL) segmenting the same image slices from all muscles for a single randomly selected participant on 2 occasions, approximately 2 weeks apart. Intra-rater reliability, as assessed using the intra-class correlation coefficient (ICC) was high, with ICCs for all muscles in excess of 0.985.

### Statistical analysis

Shapiro-Wilk tests were used to examine data normality. Demographic and clinical variables were compared between groups using independent t-tests or Pearson’s chi-square. A full-factorial, two-way General Linear Model was used to assess the effect of a between subject factor (Group) and a within subject factor (Leg) on muscle strength and volume. A priori contrasts were used to assess differences between limbs within each group. Leg was defined as affected/contralateral for participants with unilateral OA and most affected (on the basis of symptoms)/less affected for participants with bilateral OA. The test limb was randomly selected (left/right) for control participants. Effect sizes for main group effects were computed using Cohen’s d. Statistical analyses were performed using SPSS version 17.0 for Windows (SPSS Inc., Chicago, USA) with significance level set at *p* < 0.05.

## Results

There were no differences in age, height, or body mass between the hip OA and control groups. On average, participants in the hip OA group had a higher BMI than participants in the control group (*p* < 0.01) (Table 1).

**Table 1:** Participant characteristics of hip osteoarthritis and control groups

Characteristic	Unilateral hip OA *n* = 12		Bilateral hip OA *n* = 7		Hip OA *n* = 19		Control *n* = 23	
Age (years)	62.9 ± 10.0		63.0 ± 6.4		62.8 ± 8.6		58.2 ± 8.6	
Males, n (%)	3 (25%)		3 (42%)		6 (32%)		8 (35%)	
Height (m)	1.65 ± 0.09		1.69 ± 0.14		1.66 ± 0.10		1.69 ± 0.08	
Body mass (kg)	77.3 ± 14.0		77.2 ± 15.0		77.2 ± 14.0		69.9 ± 10.0	
Body mass index (kg/m^2^)	28.2 ± 3.5		27.1 ± 3.5		27.8 ± 3.5^*^		24.4 ± 3.0	
Harris Hip Score (HHS)^ab^	69.9 ± 12.9		66.2 ± 13.5		68.6 ± 12.9		99.9 ± 0.7	
	*Affected*	*Contralateral*	*Most affected*	*Less affected*	*(Most) affected*	*Contralateral/Less affected*	*Left*	*Right*
Joint space width (mm)	2.3 ± 1.1	3.5 ± 0.7	2.9 ± 0.5	3.0 ± 0.5	2.5 ± 1.0^*^	3.3 ± 0.7^*^	4.0 ± 0.5	3.9 ± 0.6
Kellgren-Lawrence grade	2, *n* = 4 3, *n* = 8	0, *n* = 4 1, *n* = 8	2, *n* = 4 3, *n* = 3	2, *n* = 5 3, *n* = 2	2, *n* = 8 3, *n* = 11	0, *n* = 4 1, *n* = 8 2, *n* = 5 3, *n* = 2	0, *n* = 12 1, *n* = 11	0, *n* = 18 1, *n* = 5

Values are mean (standard deviation) unless otherwise stated

*OA* osteoarthritis

^*^p < 0.05 hip OA compared to control group

^a^HHS scale – 0 = extreme hip problems and 100 = no hip problems; ^b^Most symptomatic hip for participants with bilateral hip osteoarthritis and randomly assigned hip for control participants; Kellgren-Lawrence grading scale – 0 = no radiographic features of hip osteoarthritis and 4 = large osteophytes

### Lower limb strength

**Fig. 2:**
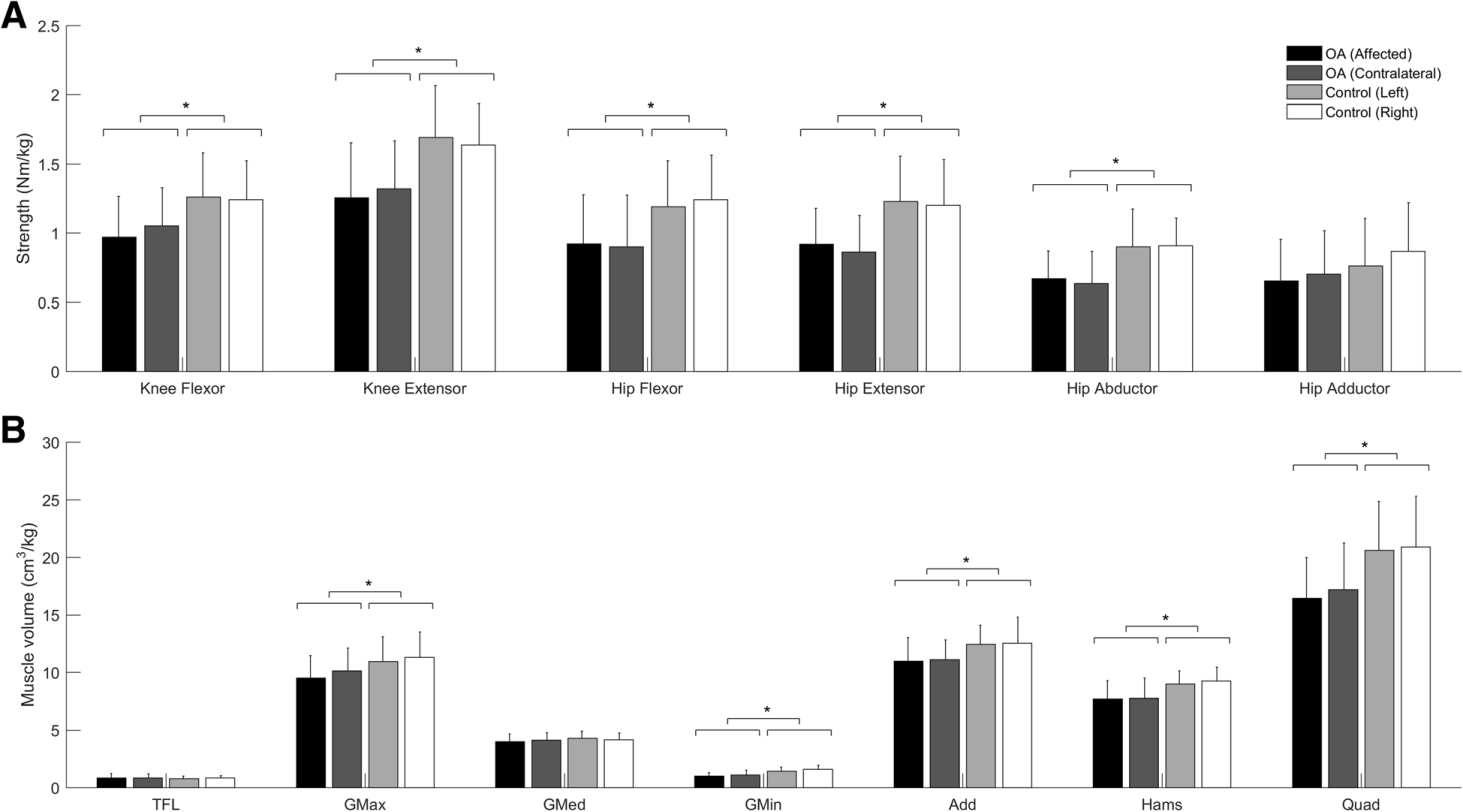
Muscle (**a**) strengths and (**b**) volumes (mean ± one standard deviation) for hip OA (*n* = 19), and control (*n* = 23) groups (TFL-tensor fasciae latae; GMax-gluteus maximus; GMed-gluteus medius; GMin-gluteus minimus; Add-adductors; Hams-hamstrings; Quad-quadriceps); Asterisk (*) indicates significant difference between hip OA and control group

No group by leg interaction effects were detected for any measure of lower-limb strength. A significant main effect of group was detected for knee flexor, knee extensor, hip flexor, hip extensor, hip abductor strength (Table 2 and Fig. 2a), but not hip adductor strength. No significant strength differences were detected between legs within each group.

**Table 2:** Summary statistics for the effect of group (hip osteoarthritis versus control) on muscle strength and volume measures

	Hip OA (mean ± SD)	Control (mean ± SD)	F, p	Mean difference (mean ± SD)	95% CI of mean difference	Effect size
*Strength (Nm/kg)*						
Knee flexors	0.977 ± 0.292	1.255 ± 0.281	9.579, 0.004^*^	0.278 ± 0.392	0.096, 0.460	0.71
Knee extensors	1.286 ± 0.344	1.664 ± 0.328	12.450, 0.001^*^	0.378 ± 0.462	0.164, 0.593	0.82
Hip flexors	0.898 ± 0.331	1.216 ± 0.314	9.866, 0.003^*^	0.319 ± 0.440	0.113, 0.524	0.73
Hip extensors	0.908 ± 0.292	1.216 ± 0.281	11.652, 0.02^*^	0.307 ± 0.392	0.125, 0.490	0.78
Hip abductors	0.662 ± 0.209	0.905 ± 202	14.34, 0.001^*^	0.244 ± 0.279	0.113, 0.374	0.87
Hip adductors	0.639 ± 0.323	0.834 ± 0.314	3.794, 0.06	0.194 ± 0.436	−0.008, 0.397	0.44
*Volume (cm* ^*3*^ */kg)*						
TFL	0.909 ± 0.324	0.816 ± 0.300	0.986, 0.327	0.094 ± 0.410	−0.285, 0.098	0.23
GMax	9.560 ± 2.336	11.119 ± 2.153	5.268, 0.028^*^	1.558 ± 2.995	0.182, 2.934	0.52
GMed	4.031 ± 0.722	4.241 ± 0.666	1.001,0.324	0.209 ± 0.911	−0.216, 0.634	0.23
GMin	1.006 ± 0.380	1.525 ± 0.352	22.048, < 0.001^*^	0.520 ± 0.484	0.295, 0.744	1.07
Add	10.827 ± 2.111	12.489 ± 1.947	7.380,0.01^*^	1.662 ± 2.668	0.420, 2.940	0.62
Hams	7.444 ± 1.548	9.117 ± 1.426	13.899, 0.001^*^	1.673 ± 1.957	0.762, 2.583	0.85
Quad	16.114 ± 4.512	20.769 ± 4.160	12.666, 0.001^*^	4.655 ± 5.701	2.001, 7.311	0.82

*Add* adductors, *CI* confidence interval, *Hams* hamstrings, *GMax* gluteus maximus, *GMed* gluteus medius, *GMin* gluteus minimus, *OA* osteoarthritis, *Quad* quadriceps, *TFL* tensor fasciae latae

^*^Significant difference between groups (*p* < 0.05)

### Hip and knee muscle volume

No group by leg interaction effects were detected for any measure of hip or knee muscle volume. A significant main effect of group was detected for GMax, GMin, Add, Hams, and Quad volume (Table 2 and Fig. 2b), but not TFL and GMed. No significant volume differences were detected between legs within each group.

## Discussion

This study compared bilateral isometric hip and knee muscle strength and hip and knee muscle volume between individuals with symptomatic and radiographic mild-to-moderate hip OA and healthy controls. Consistent with our hypothesis, individuals with hip OA tended to be weaker and have less muscle volume than those in the healthy control group. Deficts in strength were detected for the hip flexors, extensors and abductors, and the knee flexors and extensors, but not the hip adductors. Smaller muscle volumes were detected for gluteus maximus, gluteus minimus, and the adductor, hamstring and quadricep muscle groups, but not for tensor fascia latae or gluteus medius. Previous research has demonstrated generalized lower limb muscle weakness and atrophy in advanced stages of hip OA [26], and in the hip abductors in earlier stages of the disease [27]. The main and novel finding of the present study was that pervasive deficits in lower limb muscle strength and size are also present in mild-to-moderate stages of the disease process. In contrast to our hypothesis, no between-limb differences in muscle strength or volume were found in our mild-to-moderate hip OA group. Between-limb asymmetries in muscle strength and volume instead appear to primarily be a feature of advanced stage hip OA [26].

### Muscle strength and volume in individuals with mild-to-moderate hip OA

Individuals with hip OA exhibited strength deficits in the hip and knee flexors and extensors and hip abductors relative to control participants. Hip and knee muscle strength in the directions assessed was on average 22–26% lower than the control group. In general, the strength deficits in the hip OA group fall within the range reported (13–37%) in previous investigations of hip muscle strength in hip OA [12, 39]. Only hip adduction strength was not significantly lower in the hip OA group, but approached significance (*p* = 0.06) with an effect size of 0.44, which may be clinically meaningful. We therefore interpret these findings to indicate that muscle weakness in the most affected limb in mild-to-moderate hip OA tends to be generalized rather than specific to individual muscles or muscle groups and that the magnitude of weakness is similar between mild-to-moderate and advanced hip OA. The underlying cause of muscle weakness in hip OA remains unclear but could arise from decreased physical activity and/or unloading of the lower extremity during physical activity [34], perhaps driven by some combination of pain and motor dysfunction. Unresolved questions that will require further investigation concern whether muscle weakness precedes or follows the onset of hip OA, and whether weakness is a contributing cause or consequence of hip OA.

Hip and knee muscle volumes were on average 5–30% lower in individuals with hip OA across all muscle groups and individual muscles assessed, with the exception of tensor fascia latae and gluteus medius. The smaller muscle volumes in individuals with mild-to-moderate hip OA likely underpin their generalized deficits in hip and knee muscle strength, and coincide with reports of advanced hip OA [26]. In general, there was a correspondence in the amount of weakness detected at the joint level, and the atrophy of muscles that contributed to the measured strength. For example, the 22–26% lower strength of the knee flexors and extensors in the hip OA group, corresponded with 18–22% reductions in muscle volume of the hamstrings and quadriceps respectively, and suggest that muscle atrophy in hip OA is a major mechanism of underlying muscle weakness in these muscles. Our findings of lower gluteal (maximus and minimus) muscle volumes in individuals with hip OA compared to healthy controls are consistent with Zacharias et al. [27]. Further, our observations are broadly consistent with findings from a systematic review of muscle strength and size in hip OA relative to controls [26], which suggest that advanced unilateral hip OA is characterized by generalized muscle weakness and atrophy of muscles in the affected limb. Although gluteus medius had a 5% lower volume in the hip OA group, this mean group difference was not statistically significant. The tensor fascia latae muscle volume was similarly not significantly different between groups. The absence of group differences in muscle volume for these muscles could be explained by possible group differences in hip abductor muscle activation capacity, force sharing between synergistic abductor muscles and muscle quality. A further possibility is that some muscles may compensate for reduction in strength of synergistic muscles as has been observed in individuals with knee muscle pathology following anterior cruciate ligament reconstruction [40]. Indeed Grimaldi et al. [20] reported larger volumes for gluteus medius compared to healthy controls in early stages of hip pathology compared to atrophy in later stages.

### Muscle strength and volume in the affected and less-affected/contralateral limbs of individuals with mild-to-moderate hip OA

Lower muscle strength and volume did not differ significantly between-limbs in individuals with hip OA. Although 12 of 19 (63%) of our cohort had unilateral hip OA (between-limb KL grade difference ≥1), it is possible that the inclusion of 7 bilateral participants prevented asymmetries from being detected. However, a post hoc analysis of the unilateral hip OA sub-group did not reveal any clear trends to support strength or volume asymmetry (data not presented). Grimaldi et al. [20], who evaluated gluteal muscle size in individuals with mild and advanced unilateral hip OA, similarly observed no difference in muscle size between the affected and contralateral limb in the mild hip OA group. However, our observations contradict those of Zacharias et al. [27], who reported lower gluteal muscle volumes in individuals with moderate unilateral hip OA (KL grade 2: *n* = 7; KL grade 3: *n* = 13) compared to their contralateral side. When participants from Zacharias et al. [27] were dichotomized based on OA severity, only those with KL grade = 3 demonstrated atrophy in the gluteal muscles. Our cohort was comprised of 42% of individuals with KL grade = 2, which in light of the findings of Zacharias et al. [27], may suggest that muscle related asymmetry becomes more prominent with disease progression. A possible explanation for the lack of difference is muscle strength between limbs in hip OA is that rather than favouring the contralateral limb during the performance of functional tasks, individuals with mild-to-moderate hip OA unload both limbs through a reduction in overall physical activity.

Reduced muscle strength and volumes in the affected compared to contralateral limb are well documented in individuals with end-stage hip OA [14, 16, 18, 20–22]. In general, it is difficult to compare the findings from the present study to those from the literature due to differences in participant characteristics (single versus mixed sex, pre- versus post-total hip replacement), strength measurements (e.g. isometric versus isokinetic), and muscles assessed. However, findings from Zacharias et al. [27] and Grimaldi et al. [20], where a subset of lower limb muscle strength and/or muscle volumes were measured in participants with hip OA from across the disease spectrum using a consistent approach, suggest that asymmetries in strength and volume become more pronounced with disease progression. Interventions to retain bilateral muscle strength during early-middle stages of the disease therefore appear warranted in the management of hip OA. This recommendation is consistent with the evidence-based clinical practice guidelines for therapeutic exercise in the management of hip OA which recommend land-based therapeutic exercise, most notably strength training, to reduce pain, stiffness and self-reported disability, and improve physical function and range of motion [41].

### Strengths and limitations

A strength of this study was that eligibility was based on radiographic and symptomatic criteria, which minimized the well-known risk of participant misclassification [42]. There were also several limitations to the study. First, the study was not sufficiently powered to perform a sub-group analysis of unilateral and bilateral participants. A future study with a larger sample size is required to more definitively determine whether strength and muscle volume asymmetry is evident within these hip OA sub-groups. More females were recruited to the hip OA and control groups than males (hip OA: 13 female, 6 male; control: 15 female, 8 male), which may be a source of experimental bias. While the hip OA group in our study had a significantly higher BMI than controls, strength and volume measures were normalised to body mass. We chose this method as it is common and therefore facilitates comparison of findings with other studies that have used the same approach and it also has physical meaning. Strength was assessed in the present study under isometric conditions, which may not reflect muscle function during dynamic conditions including activities of daily living. It was not possible to segment boundaries for some smaller muscles (e.g. internal/external hip rotators) or muscles with insertions outside the imaged segments (e.g. iliopsoas), and thus only large hip/knee spanning muscles and muscle groups were evaluated. Further, reliability of muscle segmentation from MRI scans was established using data from a single participant. It is important for future studies to more fully elucidate the implications of reduced muscle strength and volume in mild-to-moderate hip OA for motor function and disease progression. Multiple statistical comparisons were made in the present study, which has the potential to increase the risk of type 1 error. A statistical correction was not performed due of the exploratory nature of this study [43, 44]. It is noteworthy that the hip OA cohort from the present study also exhibited reduced self-selected walking speed and altered hip joint mechanics, including lower net hip joint loading over a reduced range of hip motion for a longer proportion of the gait cycle, when walking at their preferred gait speed relative to healthy control participants [34]. These findings are consistent with an underloading hypothesis for hip OA progression, perhaps due in part to muscle weakness, which could have implications for disease progression through altered mechano-biological processes within the joint [45].

## Conclusions

The main conclusion from this study is that atrophic hip and knee muscle weakness is a distinct feature of mild-to-moderate hip OA. These strength and muscle size deficits tended to be generalized rather than localised to individual muscles and/or muscle groups in the lower limb, and have possible implications for daily function, quality of life and OA disease progression. While no evidence of between-limb asymmetry in muscle strength or volume was found in the present study, intervention early in the disease process to prevent the development of strength asymmetries that are characteristic of advanced hip OA appear warranted.

## Abbreviations

Add: Adductors
GMax: Gluteus maximus
GMed: Gluteus medius
GMin: Gluteus minimus
Hams: Hamstrings
HHS: Harris hip score
ICC: Intra-class correlation coefficient
JSW: Joint space width
KL: Kellgren-Lawrence
MRI: Magnetic resonance imaging
OA: Osteoarthritis
Quad: Quadricpes
TFL: Tensor fasciae latae
